# The Association of Low Muscle Mass With Serum Sex Hormones and Sex Hormone‐Binding Globulin

**DOI:** 10.1002/jcsm.70056

**Published:** 2025-09-11

**Authors:** Hailin Qin, Wenyong Jiao, Gui Liao

**Affiliations:** ^1^ The First People's Hospital of Hechi Hechi Guangxi China; ^2^ Department of Orthopedics Surgery The Second Affiliated Hospital of Ning Xia Medical University Yinchuan Ningxia China; ^3^ The First People's Hospital of Nanning Nanning Guangxi China

**Keywords:** appendicular skeletal muscle index, free testosterone, low muscle mass, sarcopenia, sex hormone‐binding globulin, sex hormones

## Abstract

**Background:**

The purpose of this study was to explore the associations of low muscle mass with serum sex hormones and sex hormone‐binding globulin (SHBG) using data from the National Health and Nutrition Examination Survey (NHANES).

**Methods:**

We analysed 4403 adults aged 20–59 years from the 2013–2016 NHANES. Appendicular skeletal muscle index (ASMI) was calculated as appendicular skeletal muscle (kg) divided by height squared (m^2^). Low muscle mass was defined as ASMI < 7.0 kg/m^2^ (men) or < 5.5 kg/m^2^ (women). Logistic regression was performed separately for male and female participants to assess the associations of low muscle mass with serum sex hormones and sex hormone‐binding globulin (SHBG).

**Results:**

The mean age of the participants was 38.6 years (± 11.35 years), and 46.3% of the participants were female. In men, both total testosterone (TT) (Model 3: OR = 1.003, 95% CI: 1.002–1.004, *p* < 0.001) and free testosterone (Model 3: OR = 1.007, 95% CI: 1.001–1.013, *p* = 0.022) levels correlated positively with low muscle mass. The highest TT quartile had 4.529 times the odds compared to the lowest quartile (Model 3: OR = 4.529, 95% CI: 1.927–10.645, *p* = 0.003). Each unit increase in SHBG was associated with higher odds (Model 3: OR = 1.035, 95% CI: 1.024–1.045, *p* < 0.001). The highest SHBG quartile (> 45.26 nmol/L) showed 6.442‐fold higher odds (Model 3: OR = 6.442, 95% CI: 3.134–13.242, *p* < 0.001). For women, the highest estradiol quartile (> 116 pg/mL) had 56.4% lower odds of low muscle mass than the lowest quartile (Model 3: OR = 0.436, 95% CI: 0.268–0.709, *p* = 0.004). Each unit increase in free testosterone (FT) showed an inverse association (Model 3: OR = 0.710, 95% CI: 0.605–0.834, *p* < 0.001). The highest FT quartile (> 3.70 pg/mL) had 73.7% lower odds (Model 3: OR = 0.263, 95% CI: 0.146–0.473, *p* < 0.001). Each unit increase in SHBG showed a positive association with low muscle mass (Model 3: OR = 1.009, 95% CI: 1.007–1.012, *p* < 0.001). The highest SHBG quartile (> 87.21 nmol/L) showed 5.482‐fold higher odds (Model 3: OR = 5.482, 95% CI: 2.854–10.527, *p* < 0.001).

**Conclusions:**

In women, estradiol and free testosterone levels are negatively associated with low muscle mass. In men, elevated total testosterone was unexpectedly associated with an increased risk of LMM. SHBG elevation is a consistent risk factor across sexes.

## 1 Introduction

As medical care improves, increasing life expectancy is leading to a rapidly growing elderly population. Sarcopenia—characterized primarily by loss of muscle mass, reduced muscle strength and declined physical performance—is attracting growing research attention. The prevalence of sarcopenia in community‐based populations ranges from 1% to 29% [1]. The disease is most common in older people, but it can also affect middle‐aged and younger people. Sarcopenia may result in decreased physical function, diminished ability to perform daily activities and an increased risk of falling. Several studies have suggested that sarcopenia may be associated with poor prognosis in cardiovascular, neoplastic and trauma patients [2, 3]. Sarcopenia increases the risk of hospitalization and the cost of hospital care for patients, adding to the financial burden on the health systems [4, 5]. Causes of sarcopenia may involve factors such as poor nutrition, chronic diseases, lack of exercise, medication side effects and endocrine disorders. Maintaining a certain amount of muscle mass is important for normal physiological activity in the body. Muscle mass is generally thought to be correlated with testosterone and oestrogen [6, 7]. Total testosterone levels do not fully reflect bioactive fractions. Only 2%–3% of circulating plasma testosterone is in the free state, with the rest bound to sex hormone‐binding globulin and albumin [8]. There are fewer studies on the effects of sex steroid hormones and SHBG on muscle mass. The National Health and Nutrition Examination Survey is an ongoing cross‐sectional study conducted by the National Center for Health Statistics that provides high‐quality, large sample and nationally representative data. We conducted an observational study of the 2013–2016 National Health and Nutrition Examination Survey to assess the association of low muscle mass with serum sex hormones and SHBG.

## 2 Methods

### 2.1 Study Population

**FIGURE 1:**
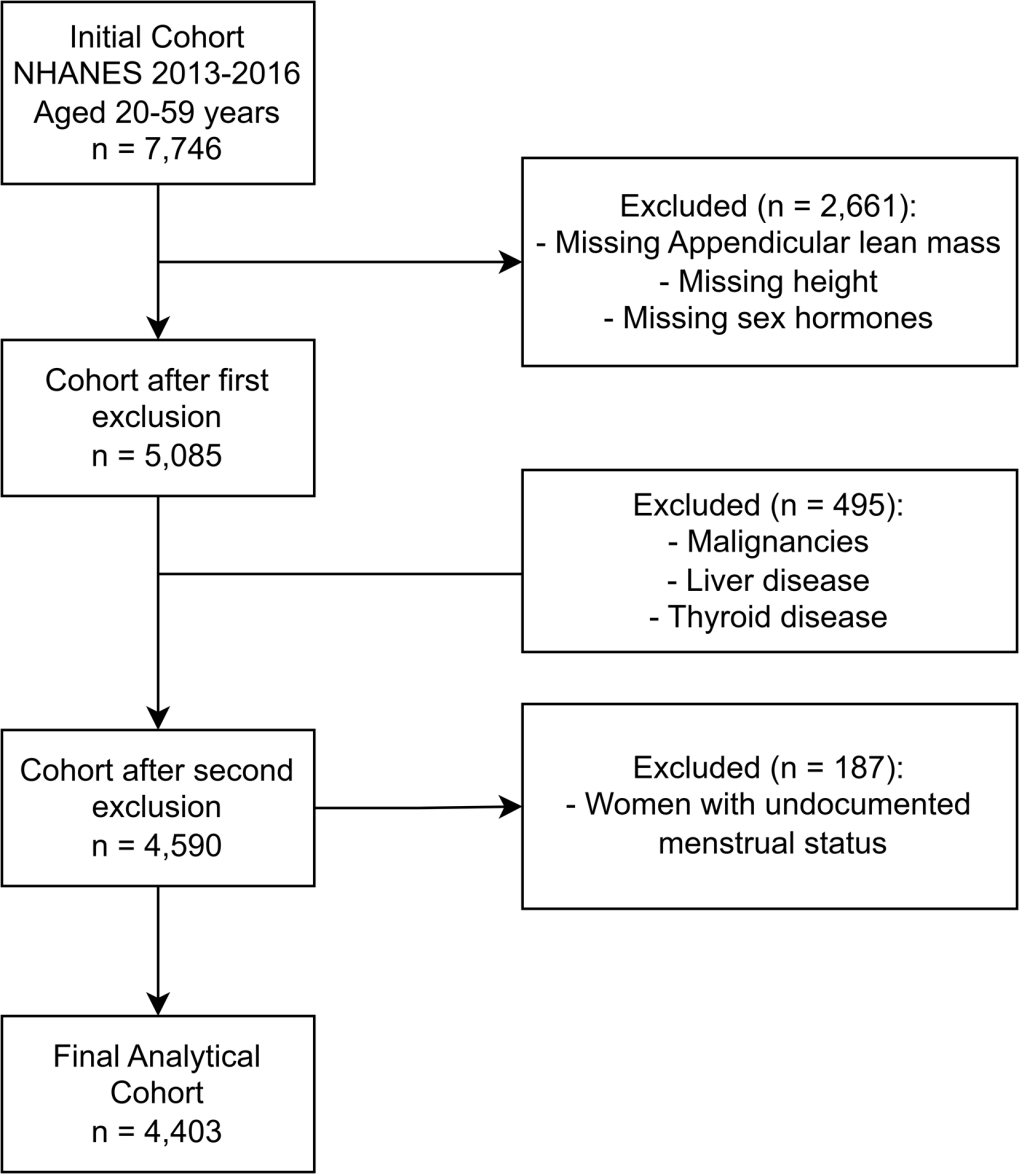
Flowchart of participant screen.

We utilized NHANES cross‐sectional data from the 2013–2016. Initially, 7746 participants aged 20–59 years were included. After applying exclusion criteria, the analytic cohort consisted of 4403 participants. Specifically, we excluded 2661 participants who lacked reliable data on appendicular skeletal muscle index, height or sex hormones; 495 individuals with malignant tumours (defined by MCQ220), thyroid disease (defined by MCQ170m) or liver disease (defined by MCQ170l); and 187 women with undocumented menstrual status (Figure 1).

### 2.2 SHBG and Sex Steroid Hormone

Serum SHBG, estradiol and total testosterone were obtained from the TST file in the laboratory data. Serum concentrations of total testosterone and estradiol were determined by isotope dilution liquid chromatography tandem mass spectrometry (ID‐LC–MS/MS). Serum sex hormone‐binding globulin was quantified by a two‐step chemiluminescence immunoassay. Detailed laboratory protocols are available in the NHANES Laboratory Methods document. Free testosterone was calculated using the Vermeulen formula [9, 10].

### 2.3 Appendicular Skeletal Muscle Index

Appendicular skeletal muscle mass was evaluated using a dual‐energy X‐ray absorptiometry (DXA). Appendicular skeletal muscle index was obtained by appendicular skeletal muscle mass divided by height squared. Low muscle mass was defined using cutoff points of 7.0 kg/m^2^ for men and 5.5 kg/m^2^ for women [11].

### 2.4 Covariate

The covariates included age, race, education level, income‐to‐poverty ratio (PIR), alcohol consumption status, total cholesterol (TC), albumin, diabetes mellitus, serum cotinine, physical activity and menstrual status (female). Physical activity levels were quantified as metabolic equivalent task‐minutes per week (MET‐min/week), calculated using the formula *MET‐min/wk = MET value × weekly frequency × duration in minutes* based on the NHANES Physical Activity Questionnaire (PAQ). Activity levels were categorized into low (< 600 MET‐min/week), moderate (600–1200 MET‐min/week) and high (> 1200 MET‐min/week) [12].

### 2.5 Statistical Analysis

Continuous variables are presented as mean ± standard deviation (SD) and were compared using Student's *t* test. Categorical variables are expressed as percentages and analysed by chi‐square test. Logistic regression models were used to examine the associations between sex hormones, SHBG and low muscle mass. The three logistic regression models were constructed as follows:

Model 1: Unadjusted.
Model 2: Adjusted for demographic factors: age, race, education level and income‐to‐poverty ratio (PIR).
Model 3: Further adjusted for clinical and behavioural covariates: alcohol consumption status, total cholesterol, albumin, diabetes mellitus, serum cotinine, physical activity and menopausal state (female).

## 3 Results

Table 1 summarizes the baseline characteristics of the participants. Significant differences were observed between males and females in race, education level, alcohol consumption, body mass index, smoking status, physical activity level, low muscle mass prevalence, albumin, cotinine and sex hormones (total testosterone, free testosterone, estradiol and SHBG). There were differences in hormone levels between males and females, so the analysis was stratified by gender.

**TABLE 1:** Characteristics of participants.

Variable	Male (*n* = 2364)	Female (*n* = 2039)	*p*
Age (year)	38.72 ± 11.44	38.42 ± 11.24	0.376
Race			0.005
Mexican American, *n* (%)	397 (16.8)	388 (19.0)	
Other Hispanic, *n* (%)	247 (10.4)	234 (11.5)	
Non‐Hispanic White, *n* (%)	824 (34.9)	669 (32.8)	
Non‐Hispanic Black, *n* (%)	443 (18.7)	425 (20.8)	
Other Race, *n* (%)	453 (19.2)	323 (15.8)	
Education			< 0.001
≤ High school, *n* (%)	1062 (44.9)	778 (38.2)	
> High school, *n* (%)	1302 (55.1)	1261 (61.8)	
Income to poverty ratio			0.120
1.3, *n* (%)	721 (30.5)	674 (33.1)	
1.3–3.5, *n* (%)	738 (31.2)	589 (28.9)	
3.5, *n* (%)	905 (38.3)	776 (38.1)	
BMI (kg/m^2^)	28.33 ± 6.01	29.25 ± 7.31	< 0.001
Drink			< 0.001
No, *n* (%)	499 (21.1)	614 (30.1)	
Moderate, *n* (%)	641 (27.1)	405 (19.9)	
Heavy, *n* (%)	1224 (51.8)	1020 (50.0)	
Diabetes			0.661
Yes, *n* (%)	156 (6.6)	127 (6.2)	
No, *n* (%)	2208 (93.4)	1912 (93.8)	
Smoking			< 0.001
Yes, *n* (%)	1096 (46.4)	606 (29.7)	
No, *n* (%)	1268 (53.6)	1433 (70.3)	
Menopausal state			
Yes, *n* (%)	N/A	527 (25.8)	
No, *n* (%)	N/A	1512 (74.2)	
Physical activity			< 0.001
Low, *n* (%)	603 (25.5)	776 (38.1)	
Medium, *n* (%)	263 (11.1)	268 (13.1)	
High, *n* (%)	1498 (63.4)	995 (48.8)	
Low muscle mass			
Yes, *n* (%)	161 (6.8)	230 (11.3)	< 0.001
No, *n* (%)	2203 (93.2)	1809 (88.7)	
Albumin (g/L)	44.63 ± 2.98	42.31 ± 3.05	< 0.001
Cotinine (ng/mL)	75.69 ± 145.32	43.12 ± 107.33	< 0.001
Total testosterone (ng/dL)	433.10 ± 182.68	24.63 ± 21.24	< 0.001
Free testosterone (pg/mL)	82.84 ± 32.04	3.05 ± 2.66	< 0.001
Estradiol (pg/mL)	24.61 ± 9.46	81.22 ± 100.46	< 0.001
SHBG (nmol/L)	36.97 ± 18.67	71.79 ± 50.05	< 0.001
Total cholesterol (mg/dL)	190.54 ± 41.07	188.14 ± 41.54	0.054

Abbreviations: BMI, body mass index; SHBG = sex hormone‐binding globulin.

**FIGURE 2:**
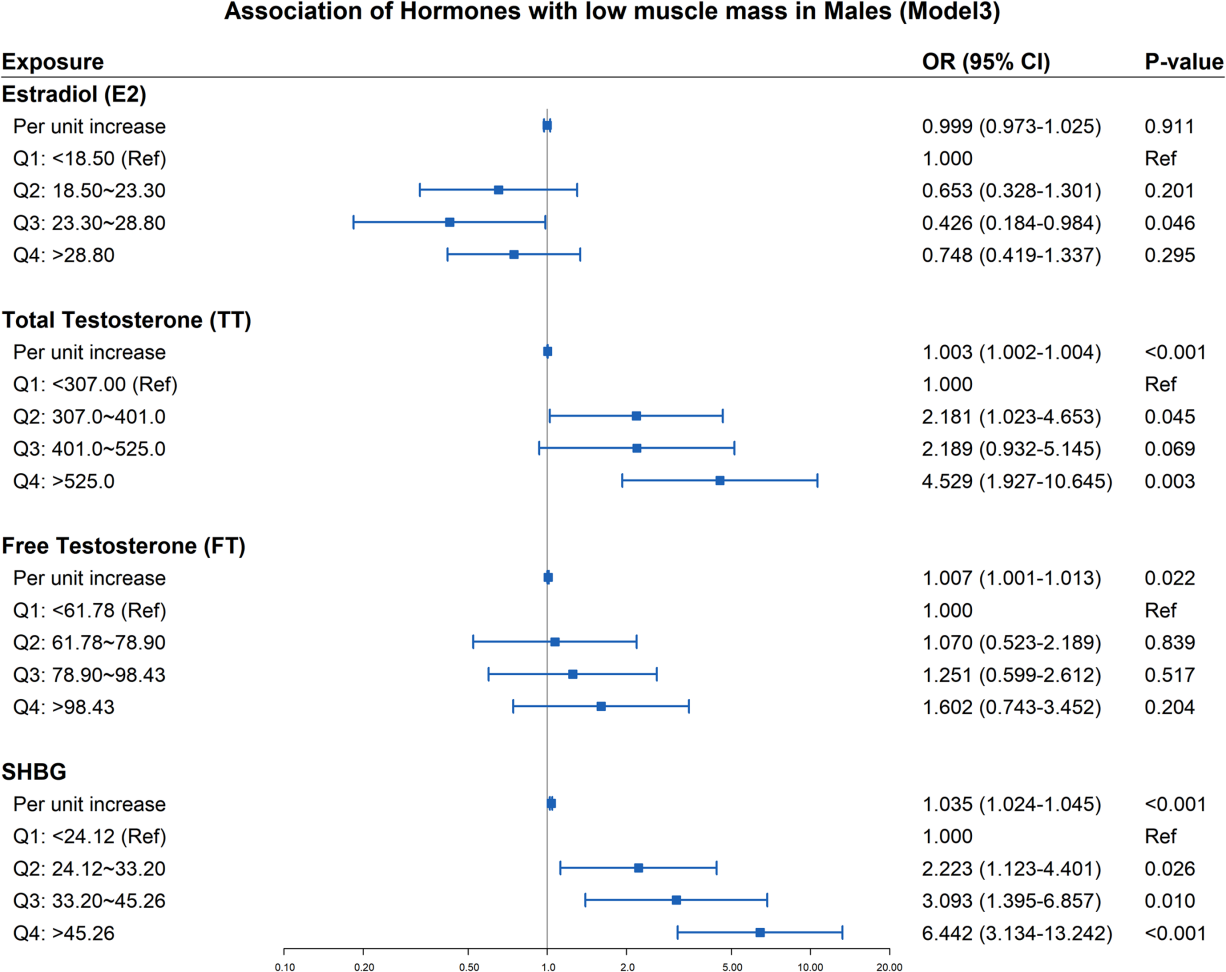
Forest plot of binary logistic regression analysis Model 3 for males. Model 3 was adjusted for age, race, education, income, drinking, total cholesterol, albumin, diabetes, cotinine and physical activity. Abbreviations: E2, estradiol; FT, free testosterone; SHBG, sex hormone‐binding globulin; TT, total testosterone.

Table 2 shows the association of sex hormones and SHBG with low muscle mass in men. In continuous models, total testosterone (OR = 1.003, 95% CI: 1.002–1.004), free testosterone (OR = 1.007, 95% CI: 1.001–1.013) and SHBG (OR = 1.035, 95% CI: 1.024–1.045) were positively associated with low muscle mass in Model 3 (all *p* < 0.05). Estradiol showed no significant association with low muscle mass in continuous models. In quartile analysis, the third quartile of estradiol (23.30–28.80 pg/mL) was associated with significantly lower odds of low muscle mass across all models (Model 1: OR = 0.422, 95% CI: 0.203–0.877, *p* = 0.023; Model 2: OR = 0.436, 95% CI: 0.202–0.939, *p* = 0.036; Model 3: OR = 0.426, 95% CI: 0.184–0.984, *p* = 0.046). In the analysis stratified by total testosterone quartiles, men in Q2–Q4 had higher odds versus Q1, though Q3 did not reach significance in Model 3 (*p* = 0.069). In the unadjusted Model 1, the highest quartile of free testosterone (Q4: > 98.43 pg/mL) showed significantly higher odds versus Q1 (OR = 1.892, 95% CI: 1.033–3.465, *p* = 0.040), but this attenuated to nonsignificance in Model 3 (OR = 1.602, 95% CI: 0.743–3.452, *p* = 0.204). Compared with the lowest quartile (Q1), SHBG demonstrated a positive dose–response association across increasing quartiles. In the fully adjusted Model 3, the highest quartile (Q4: > 45.26 nmol/L) had 6.442‐fold higher odds versus Q1 (OR = 6.442, 95% CI: 3.134–13.242, *p* < 0.001). Figure 2 shows the forest plot of the logistic regression Model 3 for males.

**TABLE 2:** Association of low muscle mass with sex steroid hormone and SHBG in male participants analysed by weighted multivariable binary logistic regression.

Exposures	Model 1		Model 2		Model 3	
	OR [95% CI]	*p*	OR [95% CI]	*p*	OR [95% CI]	*p*
E2	0.995 (0.970–1.020)	0.668	0.997 (0.971–1.024)	0.830	0.999 (0.973–1.025)	0.912
Quartiles of E2						
Q1 (< 18.50)	Ref					
Q2 (18.50–23.30)	0.663 (0.357–1.231)	0.184	0.653 (0.340–1.251)	0.186	0.653 (0.328–1.301)	0.201
Q3 (23.30–28.80)	0.422 (0.203–0.877)	0.023	0.436 (0.202–0.939)	0.036	0.426 (0.184–0.984)	0.046
Q4 (> 28.80)	0.704 (0.412–1.202)	0.189	0.719 (0.409–1.264)	0.236	0.748 (0.419–1.337)	0.295
Trend *p*		0.150		0.189		0.227
TT	1.003 (1.002–1.004)	< 0.001	1.003 (1.002–1.004)	< 0.001	1.003 (1.002–1.004)	< 0.001
Quartiles of TT						
Q1 (< 307.00)	Ref					
Q2 (307.0–401.0)	2.020 (1.006–4.057)	0.048	2.043 (0.996–4.191)	0.051	2.181 (1.023–4.653)	0.045
Q3 (401.0–525.0)	2.360 (1.082–5.144)	0.032	2.258 (1.034–4.931)	0.042	2.189 (0.932–5.145)	0.069
Q4 (> 525.0)	4.625 (2.237–9.561)	< 0.001	4.289 (2.071–8.883)	< 0.001	4.529 (1.927–10.645)	0.003
Trend *p*		< 0.001		< 0.001		0.002
FT	1.007 (1.003–1.011)	0.002	1.006 (1.000–1.011)	0.037	1.007 (1.001–1.013)	0.022
Quartiles of FT						
Q1 (< 61.78)	Ref					
Q2 (61.78–78.90)	1.055 (0.558–1.997)	0.863	1.007 (0.508–1.995)	0.984	1.070 (0.523–2.189)	0.839
Q3 (78.90–98.43)	1.392 (0.704–2.754)	0.329	1.221 (0.596–2.501)	0.567	1.251 (0.599–2.612)	0.517
Q4 (> 98.43)	1.892 (1.033–3.465)	0.040	1.469 (0.705–3.059)	0.287	1.602 (0.743–3.452)	0.204
Trend *p*		0.025		0.223		0.162
SHBG	1.025 (1.017–1.032)	< 0.001	1.034 (1.025–1.044)	< 0.001	1.035 (1.024–1.045)	< 0.001
Quartiles of SHBG						
Q1 (< 24.12)	Ref					
Q2 (24.12–33.20)	2.002 (1.093–3.665)	0.026	2.200 (1.212–3.993)	0.012	2.223 (1.123–4.401)	0.026
Q3 (33.20–45.26)	2.620 (1.271–5.397)	0.011	3.243 (1.567–6.712)	0.003	3.093 (1.395–6.857)	0.010
Q4 (> 45.26)	4.579 (2.539–8.257)	< 0.001	6.462 (3.529–11.83)	< 0.001	6.442 (3.134–13.242)	< 0.001
Trend *p*		< 0.001		< 0.001		< 0.001

*Note:* Model 1 was adjusted for none; Model 2 was adjusted for age, race, education and income; Model 3 was adjusted for age, race, education, income, drinking, total cholesterol, albumin, diabetes, cotinine and physical activity.

Abbreviations: E2, estradiol; FT, free testosterone; SHBG, sex hormone‐binding globulin; TT, total testosterone.

**FIGURE 3:**
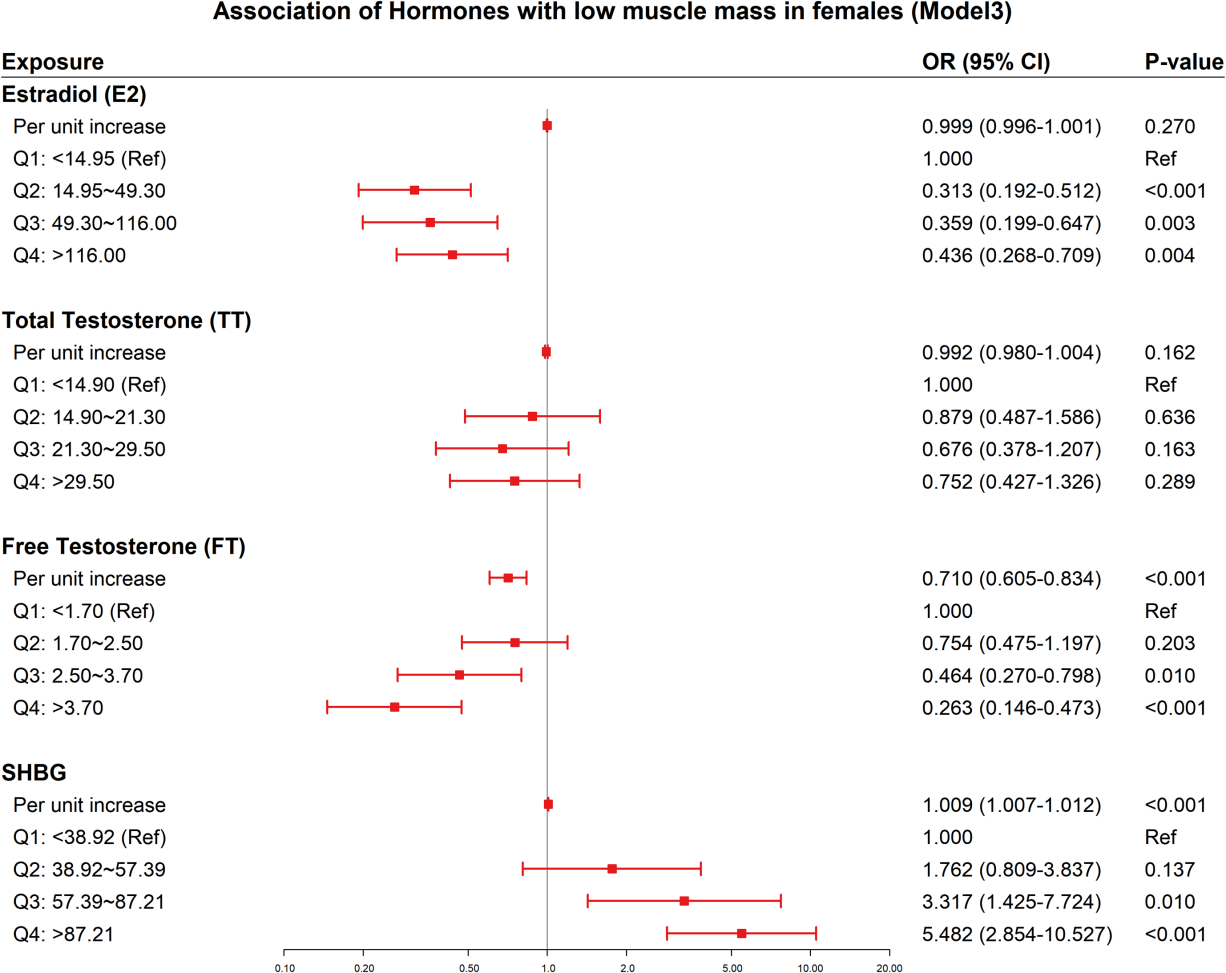
Forest plot of binary logistic regression analysis Model 3 for females. Model 3 was adjusted for age, race, education, income, drinking, total cholesterol, albumin, diabetes, cotinine, menopausal state and physical activity. Abbreviations: E2, estradiol; FT, free testosterone; SHBG, sex hormone‐binding globulin; TT, total testosterone.

Table 3 presents the results for women. In continuous models, free testosterone (FT) was inversely associated with low muscle mass (Model 3: OR = 0.710, 95% CI: 0.605–0.834, *p* < 0.001), and SHBG was positively associated with low muscle mass (Model 3: OR = 1.009, 95% CI: 1.007–1.012, *p* < 0.001). Estradiol (E2) and total testosterone (TT) showed no significant association with low muscle mass in the continuous model. In quartile analysis, versus the lowest quartile, higher quartiles of E2 (Q2–Q4) were associated with reduced odds of low muscle mass in Model 3 (Q2: OR = 0.313, 95% CI: 0.192–0.512, *p* < 0.001; Q3: OR = 0.359, 95% CI: 0.199–0.647, *p* = 0.003; Q4: OR = 0.436, 95% CI: 0.268–0.709, *p* = 0.004). For FT, the third and fourth quartiles (Q3: 2.50–3.70 pg/mL and Q4: > 3.70 pg/mL) were associated with reduced odds (Model 3: Q3: OR = 0.464, 95% CI: 0.270–0.798, *p* = 0.010; Q4: OR = 0.263, 95% CI: 0.146–0.473, *p* < 0.001). For SHBG, the third and fourth quartiles (Q3: 57.39–87.21 nmol/L and Q4: > 87.21 nmol/L) were associated with increased odds (Model 3: Q3: OR = 3.317, 95% CI: 1.425–7.724, *p* = 0.010; Q4: OR = 5.482, 95% CI: 2.854–10.527, *p* < 0.001). Significant gradient trends were observed for FT and SHBG (trend *p* < 0.001). Figure 3 shows the forest plot of the logistic regression Model 3 for females.

**TABLE 3:** Association of low muscle mass with sex steroid hormone and SHBG in female participants analysed by weighted multivariable binary logistic regression.

Exposures	Model 1		Model 2		Model 3	
	OR [95% CI]	*p*	OR [95% CI]	*p*	OR [95% CI]	*p*
E2	1.000 (0.998–1.001)	0.58	0.999 (0.997–1.001)	0.309	0.999 (0.996–1.001)	0.270
Quartiles of E2						
Q1 (< 14.95)	Ref					
Q2 (14.95–49.30)	0.494 (0.299–0.817)	0.008	0.360 (0.226–0.571)	< 0.001	0.313 (0.192–0.512)	< 0.001
Q3 (49.30–116.00)	0.618 (0.373–1.025)	0.061	0.450 (0.268–0.754)	0.004	0.359 (0.199–0.647)	0.003
Q4 (> 116.00)	0.743 (0.474–1.165)	0.187	0.538 (0.345–0.838)	0.009	0.436 (0.268–0.709)	0.004
Trend *p*		0.632		0.267		0.182
TT	0.997 (0.990–1.004)	0.342	0.993 (0.982–1.003)	0.167	0.992 (0.980–1.004)	0.162
Quartiles of TT						
Q1 (< 14.90)	Ref					
Q2 (14.90–21.30)	0.896 (0.526–1.528)	0.676	0.845 (0.495–1.444)	0.520	0.879 (0.487–1.586)	0.636
Q3 (21.30–29.50)	0.814 (0.529–1.252)	0.335	0.694 (0.408–1.182)	0.168	0.676 (0.378–1.207)	0.163
Q4 (> 29.50)	0.995 (0.626–1.582)	0.984	0.762 (0.451–1.287)	0.291	0.752 (0.427–1.326)	0.289
Trend *p*		0.918		0.299		0.268
FT	0.765 (0.665–0.880)	< 0.001	0.731 (0.633–0.843)	< 0.001	0.710 (0.605–0.834)	< 0.001
Quartiles of FT						
Q1 (< 1.70)	Ref					
Q2 (1.70–2.50)	0.838 (0.556–1.262)	0.384	0.818 (0.526–1.270)	0.351	0.754 (0.475–1.197)	0.203
Q3 (2.50–3.70)	0.519 (0.326–0.825)	0.007	0.516 (0.320–0.833)	0.009	0.464 (0.270–0.798)	0.010
Q4 (> 3.70)	0.362 (0.219–0.598)	< 0.001	0.292 (0.170–0.502)	< 0.001	0.263 (0.146–0.473)	< 0.001
Trend *p*		< 0.001		< 0.001		< 0.001
SHBG	1.008 (1.005–1.010)	< 0.001	1.008 (1.005–1.011)	< 0.001	1.009 (1.007–1.012)	< 0.001
Quartiles of SHBG						
Q1 (< 38.92)	Ref					
Q2 (38.92–57.39)	1.704 (0.857–3.389)	0.123	1.840 (0.903–3.749)	0.089	1.762 (0.809–3.837)	0.137
Q3 (57.39–87.21)	3.050 (1.475–6.309)	0.004	3.393 (1.575–7.311)	0.004	3.317 (1.425–7.724)	0.010
Q4 (> 87.21)	4.664 (2.547–8.540)	< 0.001	4.943 (2.758–8.86)	< 0.001	5.482 (2.854–10.527)	< 0.001
Trend *p*		< 0.001		< 0.001		< 0.001

*Note:* Model 1 was adjusted for none; Model 2 was adjusted for age, race, education and income; Model 3 was adjusted for age, race, education, income, drinking, total cholesterol, albumin, diabetes, cotinine, menopausal state and physical activity.

Abbreviations: E2, estradiol; FT, free testosterone; SHBG, sex hormone‐binding globulin; TT, total testosterone.

## 4 Discussion

Estradiol levels have been associated with skeletal muscle strength performance in previous studies of women [13]. Studies have shown that estradiol inhibits inflammation‐mediated release of a number of proinflammatory cytokines and promotes muscle repair through specific oestrogen receptors [14, 15]. It has also been suggested that lower oestrogen levels can increase apoptosis in skeletal muscle cells, leading to decreased muscle mass [16]. When stratifying women by estradiol quartiles, we observed significantly lower LMM risk in Q2–Q4 versus Q1 in Models 2 and 3. This association was not observed in men.

A study that included 189 female participants suggested that testosterone levels were significantly and positively correlated with appendicular skeletal muscle [17]. In our study, there was no correlation between testosterone levels and the risk of low muscle mass in women, whereas after calculating free testosterone, we found that female patients with lower levels of free testosterone had a higher risk of low muscle mass. A randomized controlled trial showed an increase in muscle mass with exogenous testosterone compared to a placebo group in a population of men with low levels of testosterone [18]. Studies on resistance‐trained men have demonstrated that changes in basal hormone levels within the normal physiologic range exerted minimal influence on muscle hypertrophy outcomes [19, 20]. Lower androgen receptor (AR) expression was detected in skeletal muscle biopsy samples from older adults [21]. In experiments where exogenous testosterone was supplied, high levels of testosterone were required to significantly improve lean body mass (LBM) and appendicular skeletal muscle mass (ASMM) in older men [22]. Exogenous testosterone demonstrated more pronounced effects on muscle maintenance in the elderly, testosterone‐deficient individuals or those with accelerated muscle loss [23]. Exogenous testosterone improved muscle mass and strength in resistance‐trained populations with testosterone deficiency [24]. Compared to previous studies, our participants were younger. This age difference may explain discrepancies in testosterone‐muscle mass associations in our male cohort versus prior research. Basal testosterone levels are much higher in young men than in women, so the effects of changes in physiologic testosterone levels on muscle mass may be masked by diet, lifestyle or physical activity, leading to the observation of opposite results to those observed in female participants [25, 26, 27]. Free testosterone may be a more important determinant of muscle mass than total testosterone levels. A study from the UK Biobank also showed that although total testosterone levels were negatively correlated with total lean body mass in men, free testosterone showed a positive correlation with it [28].

There are few studies on the effect of sex hormone‐binding globulin on skeletal muscle mass. Notably, a randomized controlled trial demonstrated that elderly men with low testosterone receiving testosterone replacement therapy (TRT) achieved significantly increased lean body mass alongside decreased SHBG levels compared with placebo [29]. Similarly, in frail elderly men, TRT was associated with lean mass gain and SHBG reduction [30]. A study of postmenopausal women reported that elevated serum SHBG was negatively correlated with muscle mass, possibly related to intramuscular fat infiltration [31]. These findings aligned with our observations. One mechanistic study found hepatic SHBG mRNA expression decreased with higher body mass index [32]. SHBG is known to regulate free sex steroid levels by binding estradiol and testosterone [33]. Elevated SHBG could reduce bioactive free testosterone delivery to muscle tissue. Recent evidence suggested SHBG actively modulated hormone signalling and liberated bioactive hormones in target tissues [33]. Our study demonstrates that elevated SHBG levels associate with increased low muscle mass risk. This finding contrasts with extensive literature linking low SHBG to heightened incidence of cardiometabolic disorders (hypertension, insulin resistance, type 2 diabetes, coronary heart disease and stroke) and neoplastic conditions [34, 35, 36, 37, 38]. The mechanism remains unclear, but abnormal levels of sex hormone‐binding globulin are associated with disease progression.

## 5 Limitations

The strengths of this study are the large sample size, which is representative of the general population in the United States, and the ability of DXA to more objectively assess the muscle mass of the participants. There are some limitations to this study. First, muscle strength is also an important indicator for the diagnosis of sarcopenia, but because there were no data related to muscle strength in the 2013–2016 NHANES database, our study focused on the loss of muscle mass. Second, this study was based on cross‐sectional data, so a causal relationship could not be determined.

In conclusion, our study elucidates that SHBG is closely related to low muscle mass and provides new insights into the endocrine mechanisms of low muscle mass.

## Ethics Statement

Data for this study were obtained from the National Health and Nutrition Examination Survey, and informed consent was obtained from all participants.

## Conflicts of Interest

The authors declare no conflicts of interest.

## Data Availability

Data used in this study are all publicly available.
